# Silver nanoparticles restrict microbial growth by promoting oxidative stress and DNA damage 

**DOI:** 10.17179/excli2020-1244

**Published:** 2020-04-15

**Authors:** Oluyomi Stephen Adeyemi, Emmanuella Oluwatosin Shittu, Oghenerobor Benjamin Akpor, Damilare Rotimi, Gaber El-saber Batiha

**Affiliations:** 1 Laboratory of Theoretical and Computational Biophysics, Ton Duc Thang University, Ho Chi Minh City, Vietnam; 2 Faculty of Applied Sciences, Ton Duc Thang University, Ho Chi Minh City, Vietnam; 3 Department of Biochemistry, Medicinal Biochemistry, Nanomedicine & Toxicology Laboratory, Landmark University, PMB 1001, Omu-Aran - 251101, Nigeria; 4 Department of Microbiology, Landmark University, PMB 1001, Omu-Aran - 251101, Nigeria; 5 Department of Pharmacology and Therapeutics, Faculty of Veterinary Medicine, Damanhour University, Egypt

**Keywords:** antimicrobial activity, medicinal biochemistry, microbial infection, nanomedicine, nanoparticles

## Abstract

Bacterial infections remain a serious health issue; hence there is a need for continuous search for improved antimicrobials. In addition, it is important to understand the antibacterial mechanism of prospective antimicrobials to fully harness their benefits. In this study, the antimicrobial action of silver nanoparticles was investigated. The antimicrobial potential of silver nanoparticles against different strains of bacteria was evaluated after which *Escherichia coli* and *Staphylococcus aureus* were selected as model for gram-negative and gram-positive bacteria respectively. Additionally, to determine mechanism of action, some biochemical assays including determination of kynurenine level, DNA fragmentation, lipid peroxidation and antioxidant status were carried out. Results showed that silver nanoparticles caused DNA damage and induced oxidative stress as reflected in elevated nitric oxide production and lipid peroxidation level. In contrast silver nanoparticles increased the antioxidant capacity *viz-a-viz*, elevated levels of total thiol, superoxide dismutase (SOD), and total antioxidant capacity (TAC) compared to untreated cells. They also initiated inconsistent alteration to the kynurenine pathway. Taken together, the findings indicate that silver nanoparticles exhibited antimicrobial action through the promotion of oxidative stress.

## Introduction

The search for newer and more effective antimicrobials is ongoing efforts. Nanoparticles (NPs) are a wide class of materials that are under 100 nm (Brayner, 2008[10]). With an increase in their popularity, they are generally used for biomedical applications such as ther-apeutics, diagnostics and drug delivery (Avalos et al., 2014[9]). Amongst some of their therapeutic potential is the ability of NPs to function as antimicrobials. Inorganic NPs, like silver NPs (AgNPs) have demonstrated such antimicrobial ability and are currently one of the most studied inorganic NPs (Feng et al., 2000[12]; Klasen, 2000[14]). They have currently been used in medical practice, such as in dental work, catheters, and the mending of burn wounds (Klasen, 2000[14]). It has also been observed that the nano-size, ionic quality and pH have a direct impact on the antimicrobial properties of AgNPs (Feng et al., 2000[12]). AgNPs have also been observed to possess lesser antibacterial ability against Gram-positive microorganisms as compared to Gram-negative microorganisms due to thinner peptidoglycan layer peculiar to gram negative microorganisms (Brayner, 2008[10]).

AgNPs are positively charged and have affinity for the negatively charged peptidoglycan layer of bacteria (Li et al., 2010[15]). When bonded, they cause their antibacterial cascade by interfering with the cell membrane and membrane transport system, hampering the cellular apparatus, inducing an increase in reactive oxygen species, and disrupting cell signaling. They also intercalate with purine and pyrimidine base sets of nucleic acids, thus irritating the hydrogen bond between parallel strands leading to DNA denaturing (Morones et al., 2005[17]). Studies have also shown the potentials of AgNPs in curbing antibiotic-resistant bacterial strains (Panda et al., 2011[18]). However, more studies are still required to fully understand the mechanism of the antimicrobial properties of AgNPs in order to fully harness its potential. To understand their mechanisms of antimicrobial action, AgNPs can be characterized and assessed against various biochemical parameters to identify their interactions in living cells. In this study, the mode of antibacterial action by AgNPs was investigated. 

## Materials and Methods

### Chemical and reagents

Silver nanoparticles were gifted by the Global Infection Unit, National Research Center for Protozoan Diseases, Obihiro University of Agriculture & Veterinary Medicine, Obihiro, Japan. Kynurenine standards, DMSO, Ehrlich reagent were products of Sigma Chemicals Co. (St Louis, Missouri, USA). Agar and broth were products of HiMedia (Mumbai, India). All other reagents were of analytical grade and used as supplied.

### Microbial culture

A total of five bacterial species (*Staphylococcus aureus, Escherichia coli, Pseudomonas aeruginosa, Bacillus subtilis and Klebsiella pneumoniae*) were used for the study. The isolates were obtained from the Department of Microbiology, Landmark University, Omu-Aran, Kwara State, Nigeria. Prior to use, the isolates first streaked on nutrient agar plates, to ascertain their purity. The pure isolates were then cultured in sterile nutrient broth and incubated at 35 °C for 24 h. All pure isolates were stored in a refrigerator at 4 °C until when needed. They were purified then streaked on nutrient agar plates and incubated for 24 h for growth at 37 °C, after which the purity of the isolates was determined.

### Determination of antibacterial and minimum inhibitory concentration

To evaluate the antibacterial potential of the AgNPs, 30 mL of sterile nutrient agar was left to cool to 45 °C, after which 1 mL of overnight culture of a respective nutrient broth cultured bacterium was added and swirled before dispensing in 20 mL quantity in a petri dish and allowed to solidify. The stock concentration (2000 µg/mL) of the AgNPs was prepared in universal bottles. Using a cork borer, three wells were bored in each plate before adding 0.2 mL of the respective AgNPs (1000 µg/mL) in a well and allowed to diffuse. Following diffusion of the AgNPs, the plates were incubated at 37 °C for 24 h. The zones of inhibition were observed and measured using a ruler and then recorded in millimeters appropriately. In each case, a control well that contained only the diluent used for preparation of the respective AgNPs was added. 

To determine the minimum inhibitory concentration (MIC) of the AgNPs, different concentrations of the AgNPs (0-1000 µg/mL) were prepared. Using the agar well diffusion method and following incubation, the lowest concentration of the AgNPs solution at which the growth of the respective isolates is inhibited (identified by zone of inhibition) was recorded as the MIC.

### Treatments of cells for biochemical assays

To accomplish the biochemical experiment, *S. aureus *and *E. coli* were selected as representative.

AgNPs only:

- The *S. aureus* was treated with AgNPs at concentrations of 700 µg/mL (MIC), 1400 µg/mL (2x MIC), 2100 µg/mL (3x MIC).
- For *E. coli*, AgNPs treatments were at concentrations of 300 µg/mL (MIC), 600 µg/mL (2x MIC), 900 µg/mL (3x MIC).

AgNPs and co-treatment with ascorbic acid:

- The treatments were as described above except for the simultaneous treatment with ascorbic acid (AA) at 1000 µg/mL. From our preliminary studies, ascorbic acid at 1000 µg/mL did not limit the growth of the bacteria isolates.

### Growth rate determination 

A growth rate curve was prepared for both *E. coli* and *S. aureus* cells, representing Gram-negative and Gram-positive bacteria. Briefly, flasks containing 150 mL of nutrient broth were prepared according to the manufacturer's instruction and labeled according to the treatments before inoculating with 500 µL of the respective bacteria culture. Following inoculation, 10 mL of the test compounds (quercetin only and/or with 1000 µg/mL of ascorbic acid) were added to the labeled flasks before incubating in a rotary incubator at 37 °C at 120 rpm. Immediately after inoculation, and every one hour interval for the first 8 h and after 24 h of incubation, 5 mL of broth was withdrawn from each flask for the measurement of optical density at a wavelength of 750 nm, using a spectrophotometer (Jenway, Staffordshire, United Kingdom). Growth rate was calculated using the formula;


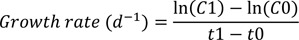


where C0 and C1 represent initial and final absorbance, respectively

t0 and t1 represent initial and final time, respectively.

### Harvesting of cells for biochemical assays

Harvesting of cells for biochemical assays was as reported previously (Dong and Cmarik, 2002[11]). Briefly, the bacteria isolate namely *E. coli* and *S. aureus* were treated in nutrient broth with quercetin only at concentrations; 0x MIC (control), MIC, 2x MIC and 3x MIC values, or in combination with ascorbic acid (1000 µg/mL). After 24-h incubation at 37 °C, the cells were harvested by centrifugation at 5,000 *g* for 10 min (model C5, LW scientific, USA). Aliquot of the supernatant was taken for kynurenine assay. The pelleted cells were washed with normal saline three times, re-suspended and thereafter homogenized and stored frozen until required for analysis.

### Biochemical assays

Biochemical determinations in cell lysates were carried out on a UV/Vis spectrophotometer (Jenway, Staffordshire, United Kingdom) where applicable. Determination of kynurenine in bacteria culture suspension was performed according to established protocol (Adeyemi et al., 2017[5]). Nitric oxide concentration was measured as the nitrite level according to the method described elsewhere (Adeyemi and Sulaiman, 2014[8]). Total antioxidant capacity (TAC) of cell lysates was determined as described previously (Adeyemi et al., 2018[3]). Total protein concentration was estimated according to a method described previously by Gornall et al. (1949[13]) with slight modification. Potassium iodide was added to the biuret reagent to prevent precipitation of Cu^2+^ ions. The total thiol level was determined by the method described elsewhere (Adeyemi and Orekoya, 2014[7]). Lipid peroxidation was estimated as malondialdehyde (MDA) using the method described previously (Adeyemi et al., 2017[4]). DNA fragmentation was determined using the diphenylamine (DPA) assay as described elsewhere (Adeyemi et al., 2017[5]). The superoxide dismutase (SOD) activity was also determined using previously reported protocol (Misra and Fridovich, 1978[16]).

### Statistical analysis

Results were analyzed by using one-way analysis of variance (ANOVA) on a GraphPad Prism 6 (GraphPad Software Inc., San Diego, California, USA). Data are expressed as mean of three replicates ± standard error of mean (SEM). Comparisons among group mean values were performed by Tukey's post-hoc test and *p*< 0.05 was considered to indicate a significant difference.

## Results

### Antibacterial determination

**Figure 1:**
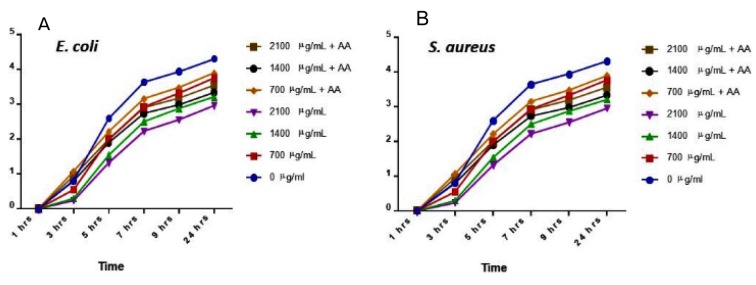
Microbial growth curve for *E. coli* (A) and *S. aureus* (B) at 24 hrs treatment with silver nanoparticles (AgNPs) and/or ascorbic acid (AA). Data are presented as mean of duplicates ± standard error of mean (SEM).

**Figure 2:**
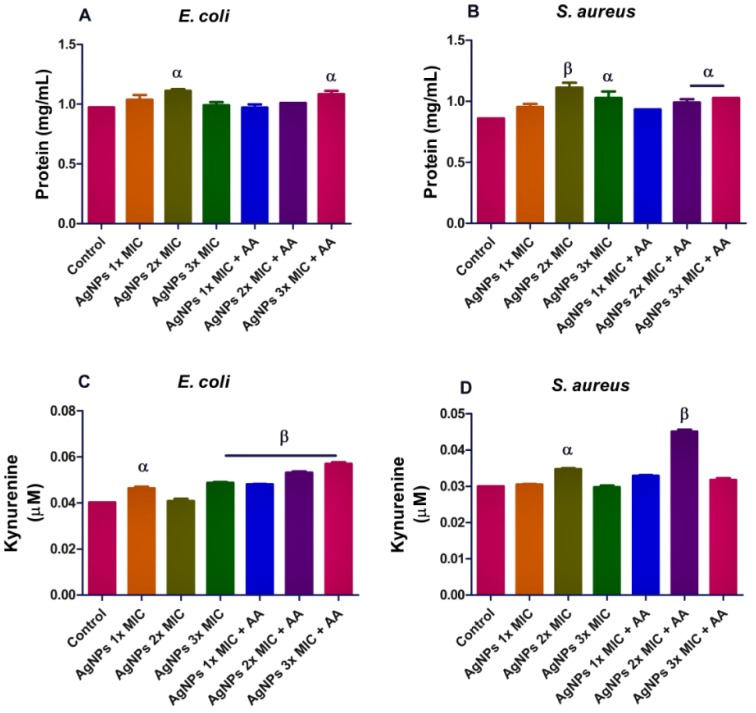
Protein (A and B) and kynurenine (C and D) levels in bacterial isolates following exposure to silver nanoparticles (AgNPs) and/or co-treatment with ascorbic acid (AA). Data are presented mean of duplicates ± standard error of mean (SEM). α is significant at p<0.05 versus control, β at p<0.01 control or AgNPs 2x MIC (kynurenine in *S. aureus* - D).

AgNPs showed antibacterial activity against all the strains (Table 1(Tab. 1)). MIC analysis also showed considerable antibacterial effect of AgNPs (Table 2(Tab. 2)). The growth rate curve of *E. coli* and *S. aureus* showed evidence of bactericidal effect of AgNPs (Figures 1(Fig. 1) and 2(Fig. 2)). Treatment with DMSO and ascorbic acid showed no antimicrobial effect. The ascorbic acid was included to determine whether oxidative stress by AgNPs was culpable in the antibacterial action.

### Biochemical assays

To determine if oxidative stress contributes to the antibacterial action of AgNPs, we included an antioxidant (ascorbic acid - AA) in the assay medium. Results showed that AgNPs and its co-exposure with AA caused an increase in the protein content of the microorganisms after 24-hour incubation, p<0.05 (Figure 2A and B(Fig. 2)). In order to evaluate whether, the activation of kynurenine might be involved in the action mechanism of AgNPs, we determined the level of kynurenine in culture supernatant. Results showed inconsistent effect of AgNPs on the kynurenine pathway in both microorganisms, p<0.05. (Figure 2C and D(Fig. 2)). 

**Figure 3:**
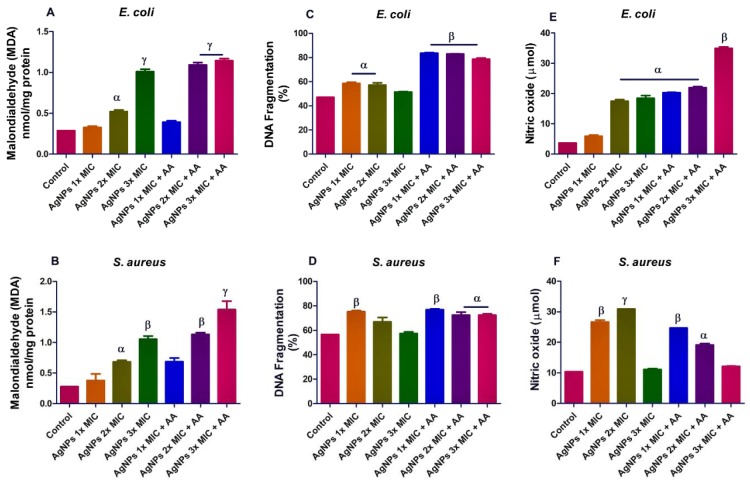
Effects of AgNPs and/or co-treatment with ascorbic acid on bacterial isolates; lipid peroxidation (A and B)], DNA damage (C and D) and nitric oxide level (E and F). Data are represented as mean of duplicates ± standard error mean (SEM). α is significant at p<0.05 versus control, β at p<0.01 versus control or AgNPs 3x MIC (nitric oxide in *E. coli* - E), and ɣ at p<0.0001 versus control or AgNPs 3x MIC (malondialdehyde in *S. aureus* - B).

Furthermore, we assayed for MDA level in order to evaluate if AgNPs caused lipid peroxidation. Our results showed evidence of lipid peroxidation in both microorganisms following exposure to AgNPs (Figure 3A and B(Fig. 3)). In addition, we determined DNA damage in the bacterial isolates and found that the combination of AgNPs and ascorbic acid caused higher incidence of cellular DNA damage than the effect of AgNPs alone. This was more evident in *E. coli*, p<0.05 (Figure 3C and D(Fig. 3)). Moreover, the AgNPs exposure led to increased nitric oxide production in the bacteria isolates (Figure 3E and F(Fig. 3)). Taken together, the findings suggest that AgNPs might have caused oxidative stress in the bacterial isolates.

**Figure 4:**
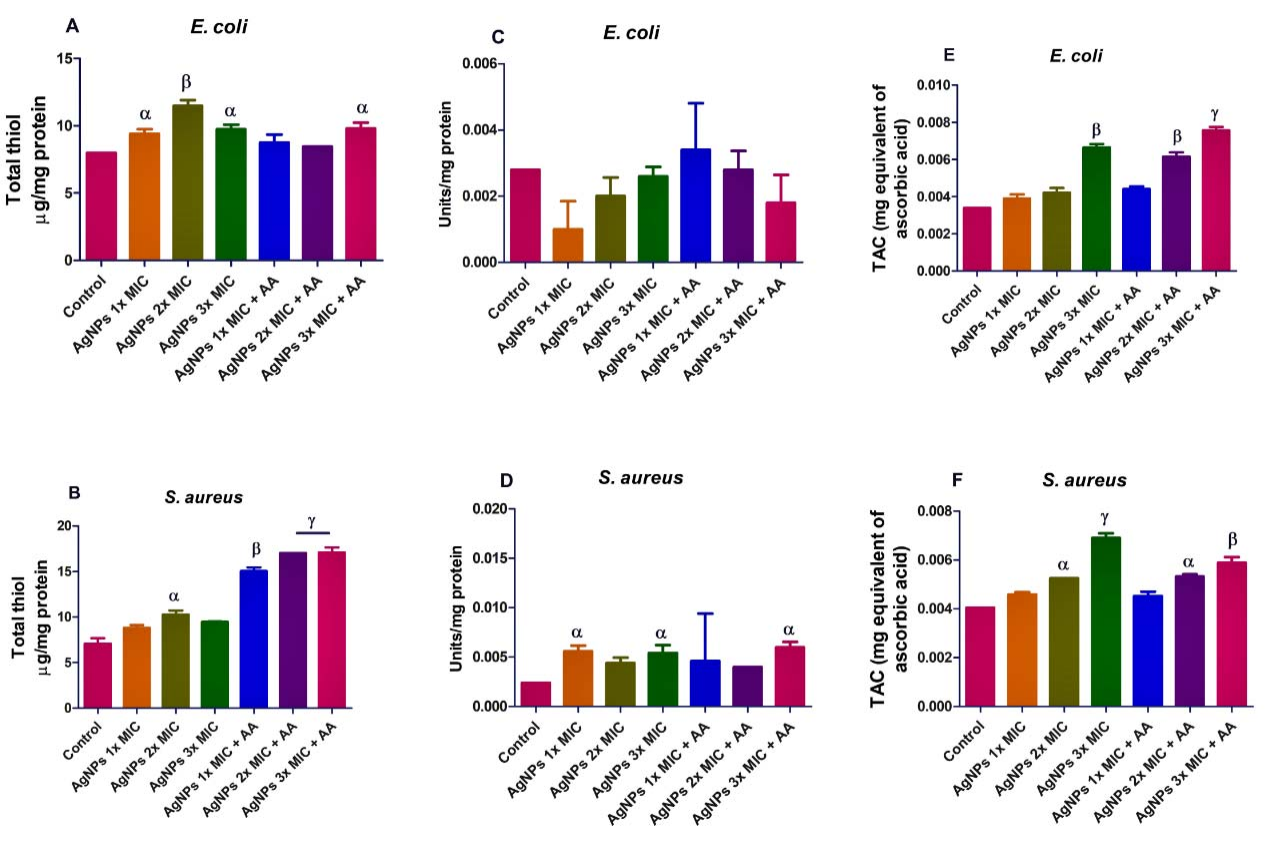
Effects of AgNPs and/or co-treatment with ascorbic acid (AA) on bacterial isolates total thiol (A and B), superoxide dismutase (SOD) (C and D), and total antioxidant capacity (TAC) (E and F). Data are presented as mean of duplicates ± standard error mean (SEM). α is significant at p<0.05, and β at p<0.01 versus control and/or AgNPs 1x MIC (total thiol in *S. aureus* - B), ɣ at p<0.0001 versus control and/or AgNPs 3x MIC (total thiol in *S. aureus* - B).

However, and in contrast to its capacity to induce oxidative stress, the AgNPs exposure led to a higher total thiol level in *E. coli* and *S. aureus* when compared with the control, p<0.05 (Figure 4A and B(Fig. 4)). In like manner, the SOD activity was elevated in *E. coli* and *S. aureus* (Figure 4C and D(Fig. 4)). This may likely be in response to AgNPs-induced oxidative stress noting that SOD is an inducible enzyme. Also, the TAC in the bacterial isolates was increased (p<0.05) after exposure to AgNPs and/or ascorbic acid (Figure 4E and F(Fig. 4)), suggesting probable antioxidant properties.

## Discussion

The biomedical application of AgNPs has been attracting huge enthusiasm. This is because of their alluring and exceptional properties such as the nano-size and large surface area to volume ratio as well as their characteristic antimicrobial properties. Their broad-spectrum bactericidal impacts are well reported (Feng et al., 2000[12]). Diverse investigations have demonstrated the ability of AgNPs to cross the bacterial layer and infiltrate the cell, thus distorting the cellular architecture, and causing cell death (Yan et al., 2018[22]). Our results showed that the silver nanoparticles had promising antimicrobial activity against *S. aureus* and *E. coli*. The microbial growth curves show that AgNPs possesses broad antimicrobial properties as it inhibited the growth and replication of both *E. coli* and *S. aureus*. 

In the present study, AgNPs raised the level of protein content in the bacterial isolates. This might be due to increased protein synthesis as a consequence of AgNPs-imposed stress. Meantime, alteration in the kynurenine levels following AgNPs exposure in both *S. aureus* and *E. coli*, may indicate an effect on tryptophan metabolism. For example, oxidative stress has been linked with the oxidative degradation of tryptophan to kynurenine (Adeyemi et al., 2019[2]). So if as a result of oxidative stress, the kynurenine pathway becomes activated, consequently the local concentration of L-tryptophan would reduce thereby, making this aromatic amino acid a limiting growth nutrient for the bacteria. In the present study, AgNPs caused oxidative stress as reflected in the increased levels of lipid peroxidation. The finding may indicate the involvement of oxidative stress in the antimicrobial action of AgNPs against *E. coli* and *S. aureus*. The increased levels of nitric oxide may also indicate nitrosative stress which could lead to the covalent binding of DNA, proteins and lipids (Wink and Mitchell, 1998[21]). This finding is consistent with previous reports that have shown that AgNPs caused oxidative stress *in-vivo* and *in-vitro* (Avalos et al., 2014[9]; Adeyemi et al., 2017[5]; 2019[6]). Furthermore, DNA damage in the bacterial isolates after AgNPs exposure might not be unconnected with the capacity of the nanoparticles to promote oxidative stress as observed in this study. More so, the present findings are consistent with previous reports of AgNPs-induced DNA damage and oxidative stress (Adeyemi et al., 2017[5]; 2019[2]; Sulaiman et al., 2015[19][20]). Furthermore, a previous study (Feng et al., 2000[12]), had shown that AgNPs caused DNA fragmentation in bacteria and this eventually led to cell death. Moreover, the elevated level of SOD following exposure to AgNPs may further support that oxidative stress might be culpable in the action mechanism of AgNPs against the bacterial isolates. SOD is an inducible enzyme in response to oxidative stress; therefore, it is plausible that the elevated SOD activity was a response to induction of oxidative stress by AgNPs. In addition, the increase in TAC and level of total thiol following exposure to AgNPs might be due to an adaptive mechanism by the bacterial isolates to offset the oxidative stress imposed by the treatment. Although, it is not uncommon for compound to have opposite chemical properties, AgNPs in this study showed a strong pro-oxidant and mild antioxidant potential. In our previous investigations, we have reported that AgNPs caused elevation in levels of rat plasma and liver thiol groups (Adeyemi and Faniyan, 2014[1]; Adeyemi et al., 2018[6]). Meanwhile, addition of ascorbic acid does not appear to reverse or improve the oxidative stress and DNA damage caused by AgNPs. Infact, in some instances, addition of ascorbic acid aggravated the effect of AgNPs on oxidative stress parameters and DNA damage.

## Conclusion

With various reports on the antibacterial properties of AgNPs, it is important to investigate the antimicrobial action mechanism of the nano-sized particles. Our data support that the antimicrobial properties of AgNPs in *E. coli* and *S. aureus* might not preclude the alteration of redox status, DNA damage as well as activation of the kynurenine pathway. Additionally, data showed that AgNPs might elicit mild cellular antioxidant response. 

## Conflict of interest

The authors declare that they have no conflict of interest.

## Acknowledgements

Appreciate the Ton Duc Thang University, Ho Chi Minh City, Vietnam. Also, the authors wish to thank the laboratory staff in the Landmark University Departments of Biochemistry and Microbiology for their technical support. Bruce Barron and Carey are appreciated for review and editorial assistance.

## Figures and Tables

**Table 1:**
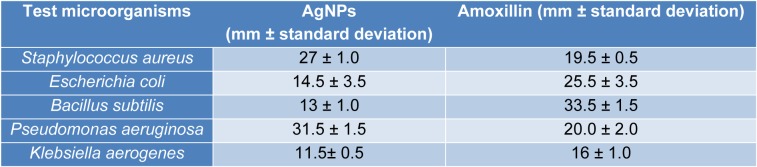
Zone of Inhibition of silver nanoparticles (AgNPs) on various microorganisms

**Table 2:**
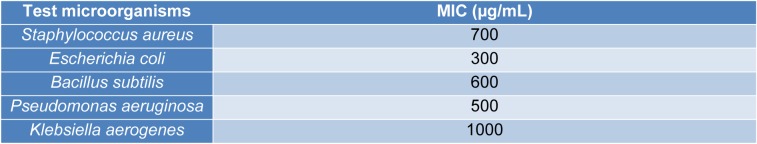
Minimum inhibitory concentration of silver nanoparticles (AgNPs)
